# Supplementary material for: Are carbon emissions trading and green financial instruments synergistic? -Comprehensive quantitative research based on content analysis
Source: PLoS One. 2024 Mar 7;19(3):e0298601. doi: 10.1371/journal.pone.0298601 (PMC10919601; doi:10.1371/journal.pone.0298601)
Supplement: S1 Data — (ZIP) [file pone.0298601.s001.zip › Data/地级市Z_F工作报告.zip!/╕╩╦α/┬ñ─╧╩╨/└┤╫╘╣1⁄2╓┌║┼í╛┬φ┐╦╩2╛▌═°í┐_2008.docx]

各位代表：

现在，我代表市人民政府向大会作工作报告，请予审议，并请各位政协委员和列席人员提出意见。

一、2007年工作回顾

刚刚过去的2007年，是全市人民在市委的坚强领导下，奋力拼搏、以干克难、艰辛创业的一年，是全市经济社会发展取得丰硕成果的一年，是解决民生、改善民生，人民群众得实惠最多的一年。一年来，我们按照市第二次党代会和二届一次人代会的部署，全面贯彻落实举特色旗、走富民路、建小康市的发展战略，全面推进生态、文化、和谐、富庶陇南建设，着力落实“一纲九目”工作重点，全面超额完成了全年各项目标任务，实现了“两个高于”、“五个大幅增长”的目标。预计全市生产总值达到112亿元，比上年增长12.8%；一、二、三产业增加值分别达到30.7亿元、40.43亿元、40.87亿元，分别比上年增长5.9%、17%、14%；城镇居民人均可支配收入达到7650元，比上年增长8.2%；农民人均纯收入达到1600元，比上年增长9.3%，是近年来农民增收最多的一年。

1、坚持发展抓项目不放手，项目建设取得重大突破。我们突出项目建设这个总纲，将2007年确定为重大项目突破年，全面落实领导干部包抓项目产业“五个一”责任制，各级领导、各行各业全力抓项目，建成了一批带动经济增长、财政增收、群众得实惠的大项目、好项目。去年共争取各类投资项目310项，投资11.38亿元，是争取国家和省上项目最多的一年。我们制定招商引资优惠政策，优化招商引资环境，加大招商引资工作力度，创新招商引资方式，去年共签约招商引资合同项目180个，拟引资金76.95亿元，到位资金24.78亿元，分别比上年增长147%、113%。去年全市开工建设各类项目894项，实施投资5000万元以上的项目79项，其中投资过亿元的项目38项，完成全社会固定资产投资50.3亿元，比上年增长40%，拉动经济增长7.5个百分点。

2、农业特色产业开发取得新进展，产业化经营水平明显提升。我们紧紧围绕发展现代农业，加快农业特色产业发展步伐，召开了第二届农业特色产业发展大会，建立了市级领导包抓特色产业责任制，市级财政安排600万元的专项资金扶持农业特色产业，广大群众以前所未有的积极性发展农业特色产业。去年全年新发展农业特色产业面积256.52万亩，其中补植新种核桃115万亩。花椒、油橄榄种植面积和产量跃居全国、全省第一。核桃面积和产量居全省第一、全国第四。建成市级以上龙头企业20个，实现农产品加工增加值4.05亿元，比上年增长62.5%；新建、完善农产品专业市场17个，发展农民专业经济合作组织121个。“一注册三认证”得到加强，无公害农产品产地认定141万亩，绿色食品产地认定14万亩，无公害产品认证42个，注册农产品商标50个，推进了农业标准化生产进程。创办农民科技学校981所，培训农民40.83万人。市上多次组织参加全国性大型节会，我市油橄榄、茶叶等特色农产品获得由国家农业部、科技部等部委颁发的后稷奖等36项大奖，特色农产品品牌效应进一步扩大。预计全市农业特色产业总产值达到23.6亿元，农民人均特色产业收入有了较大提高。粮食直补等各项支农惠农政策全面落实，去年全市粮食产量达到89.5万吨，比上年增长17%，创历史最高水平。

3、交通大建设深入实施，基础设施建设明显加快。集全市智慧，举全民之力开展了公路交通大建设，交通面貌发生了可喜变化。全市新建改建农村公路479条3994公里，打通断头路、出口路、联网路42条579公里，整修养护农村公路1235条8521公里。特别是西汉水流域六县区的各级组织和广大干部群众众志成城、以干克难、不等不靠、艰苦创业，不到一年时间，新建公路桥31座，改建公路569公里，打通了全长286公里，惠及22个乡镇、305个村、25万群众的龙昌公路，铸就了彰显陇南人民智慧和力量的昌河坝精神。掀起了新一轮城市建设高潮，市区建设取得重大进展，东江、钟楼滩新区开发建设、旧城改造全面展开，市区东出口、北出口畅通工程已经竣工，长江大道旧城区段基本建成铺油，莲湖文化广场、建民大桥等项目和南北两山绿化、双十园建设进展良好；八县县城建设和改造稳步推进，徽县、两当等县城的新区建设取得突破性进展，城镇化建设进程进一步加快。加强能源建设，武都330、西和330千伏送变电工程开工建设，“户户通电工程”竣工通电，解决了1.2万户的用电问题，户通电率达到95%；新建沼气池1.96万口，发展太阳灶1.8万台；积极实施生态建设、农田水利建设、农村饮水安全和通讯建设，完成生态林30.63万亩，解决了6.08万人的饮水安全问题；全市移动电话、固定电话、互联网用户分别新增10万户、5.3万户、7000户，群众生产生活条件进一步改善。

4、实施工业强市迈出新步伐，工业发展的规模质量效益有较大提高。召开了首届工业发展大会，确立了加快工业科学发展的思路和目标任务，制定了工业发展的一系列政策措施，工业经济发展步伐明显加快。去年实施各类工业项目200项，完成投资17.84亿元。宝徽集团6万吨锌冶炼二期技改项目、金口坝等11个水电站建成投产，麒麟寺、苗家坝、椒园坝等电站建设进展顺利，成州集团10万吨锌冶炼技改扩建项目进展较快。以金徽酒业、油橄榄深加工、长城果汁等为主体的绿色食品工业有了新的发展，礼县、文县石材加工、礼县乳化炸药等工业项目开工建设。工业聚集区建设步伐加快，西成经济开发区跨入全省34个省级开发区之列。去年全市规模以上工业预计完成增加值30.1亿元，比上年增长21.6%。全市纳税200万元以上的企业由上年的65户增加到76户，工业对财政收入的贡献达到72%。全市非公有制经济快速发展，预计全年非公经济实现增加值44.93亿元，比上年增长22.47%，占生产总值的比重达到40.1%。节能减排、环境保护、矿产资源开发管理秩序整顿规范得到有效加强，节能减排任务全面完成，主要能耗指标低于全省水平，关闭非法采矿点（硐）281个，矿产资源整合取得实质性进展，全市7个矿区的矿权由19个减少为7个，矿产资源开发管理秩序明显好转，资源利用率和集约化程度明显提高。

5、旅游开发实现新突破，第三产业发展提速。召开了第二届旅游发展大会，积极推进陕甘川毗邻地区大旅游经济圈建设，与三省十二方的旅游区域合作不断深化；高度重视旅游规划编制，全市旅游总体规划通过省级评审；加大旅游景区开发和以宾馆饭店为主的旅游基础设施建设力度，全力打造10个名牌景点，成县西狭、武都万象洞、宕昌官鹅沟、康县阳坝等景区被国家旅游总局命名为4A级景区，成州国际大酒店被省旅游局评定为四星级宾馆，东诚大酒店等7家宾馆分别被评定为三星或二星级宾馆；全方位、多形式开展旅游宣传推介，成功举办了成县西狭旅游文化艺术节、西和乞巧文化节、康县阳坝采茶节、宕昌官鹅沟旅游文化节等4个旅游节会，提升了陇南旅游景区的知名度和吸引力，旅游业呈现出迅速崛起的强劲势头，去年全市接待游客250万人次，旅游综合收入达到3.5亿元，分别比上年增长47.3%、33.6%。旅游在第三产业发展中的辐射带动作用不断增强，市场体系建设明显加快，商贸流通日益活跃，城乡群众消费结构升级，预计全市实现社会消费品零售总额25亿元，比上年增长15%。

6、扶贫开发力度加大，新农村建设稳步推进。召开了全市扶贫开发大会，按照产业开发、整村推进、劳务输转、移民搬迁、科技扶贫的五条路子，扎实推进扶贫开发，投入财政扶贫资金1.17亿元，扶贫贴息贷款资金8458万元，世行贷款809万元，社会帮扶资金3658万元，完成扶贫整村推进115个，整流域推进项目8个，输转劳务59.63万人，创收24.29亿元，比上年增长24.95%；向外移民1.1万人；全市减少贫困人口5.5万人。动员全社会力量参与扶贫开发，市、县直单位、企业各包抓一个贫困村，将各级领导干部的联系帮扶村全部调整到最偏远山区的最贫困村，做到了对2106个贫困村的全覆盖，市、县级1000多名领导干部深入贫困村开展了结穷亲、解民忧、帮民富、受教育、长德才、求发展、促和谐帮扶活动，实施帮扶项目820项，投入各类资金和捐物折款1578.68万元，产生了显著的效益。召开了全市新农村建设现场会议，狠抓省市县列试点村建设，新农村建设取得明显进展。

7、财政收入迈上新台阶，公共财政职能进一步发挥。加大税收征管力度，强化非税收入管理，全市大口径财政收入达到16.12亿元，比上年净增4.54亿元，增长39.2%，增幅居全省第二；市县级财政收入达到5.77亿元，比上年净增1.88亿元，增长48.2%，增幅位居全省第一。财政收入的快速增长，有效缓解了自我发展能力不足的问题。完成财政支出42.97亿元，比上年增长38.5%，增支11.97亿元。大幅度增加公共财政支出，为全市干部职工每月增加津贴补贴416元，在城市低保、农村低保、新型合作医疗、农村“两免一补”、“五保户”供养、教育、粮食直补、扶持农业生产、社会事业发展等关注民生办实事方面的投入达到13.3亿元，比上年增长54.02%，重点支出和民生投入之多是从来没有过的，广大人民群众共享了经济社会发展带来的实惠和成果。金融机构年末存款余额达到162.96亿元，贷款余额达到68亿元，分别比年初增长19.75%、30.21%，有力地支持了全市经济建设和社会事业发展。

8、各项改革不断深化，对外开放水平明显提升。184户国有企业改制和粮食流通体制改革基本完成，一批企业通过改制焕发出新的生机与活力，供销企业总体上在全省率先实现扭亏增盈。以校长聘任制、任期制和教师全员聘任制为重点的教育人事制度改革取得实质性进展，农村综合改革、行政审批制度、财政金融、投资体制、国有资产监管和文化、卫生、科技等各项社会事业改革有序推进。坚持以开放促发展，走出山门，打破封闭，组团参加了济南中国国际农产品交易会、陕西杨凌“农高会”、甘肃省第二届林果花卉展交会、广交会、科博会、西博会、兰洽会等重要节会，市县（区）领导多次亲自带队走出去招商引资，成功举办了陇上江南冬季露地蔬菜博览会、明前茶鉴品会、首届采茶节、首届花椒节、首届核桃节、油橄榄产品展示会等大型节会，有效地聚集了人流、物流、信息流，最大限度地提升了人气，提升了陇南的知名度和影响力。与中国林科院、西北农林科技大学、省科学院、农科院、社科院等建立了科技开发协作关系；聘请国内外知名专家学者当顾问，来陇南培训技术人员，指导产业开发；发展外向型经济打开新的局面，建成外贸出口企业4个，完成外贸出口1500万美元，比上年增长了10.2倍，增幅居全省第一。陇南全方位大开放的格局正在形成。

9、关注民生办实事取得实效，社会事业发展步伐加快。去年我们倾注全力改善民生办实事，市委、市政府确定的13件实事基本完成。新型农村合作医疗参合人数达到210.26万人，参合率达到86.3%；城镇居民医疗保险参保率达到76.5%；全市乡镇基层卫生院人员工资实现了由财政全额供给，县区和市级医疗机构床位补贴实现了翻番，10%的济困病床覆盖市县(区)公立医院；城镇居民最低生活保障标准提高了10%，发放城市保障金5238.71万元，基本实现了动态管理下的应保尽保；全面启动了农村居民最低生活保障制度，保障对象人数23.67万人，占农村绝对贫困人口的80%，发放保障金4715.3万元；养老、医疗、工伤保险覆盖面不断扩大；解决城镇零就业家庭成员就业497人，城镇新增就业人数1.01万人；五保户保障标准提高到每人每年1200元，发放保障金1513.7万元；全市村干部报酬标准由上年的每人每年1200元提高到1600元；清欠2679名农民工的工资218.5万元；城市低收入家庭享受廉租住房保障的达到1524户、3934人。解决大中专毕业生就业3231人。教育、科技、文化等各项社会事业全面发展，召开了全市教育发展大会，制定了加快教育发展的政策措施，为26.93万名中小学生实行了“两免一补”，礼县、康县“两基”攻坚通过省上验收；建成寄宿制学校68所，实施农村中小学危房改造项目134项；录用教师2812人，教师队伍得到充实加强；教育质量进一步提高，普通高考上线人数达到6822人。科技创新能力得到增强，建立企业科技研发中心16个，培育科技创新和科技示范企业21户，实施重点科技项目44项，获得科技成果30项，下派科技特派员1459名。县乡医疗卫生基础设施不断完善，建成县级医疗机构2个，乡镇卫生院35个。文化陇南建设迈出新步伐，大型文化活动异彩纷呈，开展了科技文化卫生“三下乡”、春节社火调演和“千台大戏送农村”活动，非物质文化遗产保护工作走在全省前列。广播电视和体育事业不断进步，45个乡镇225个村的20万群众看上了有线电视。人口低生育水平保持稳定，人口自然增长率控制在6.8‰以内，全面完成了人口计划，礼县计划生育工作扭转了被动局面，徽县被国家人口和计划生育委员会正式命名为国家级计划生育优质服务县。以民兵预备役工作为中心的国防后备力量建设、国防教育、双拥工作、民族宗教、统计审计、气象物价、地震预防、残疾人事业等都取得了新的成绩。

10、民主法制建设得到加强，团结和谐的局面不断巩固。坚持依法行政，民主行政，接受人大法律监督、政协民主监督的制度和程序不断健全完善，办理人大代表建议和政协委员提案229件、256件。继续实施全面推进依法行政五年规划，政府依法行政的水平不断提高。深入推进政务、村务、厂务公开，基层民主进一步扩大。认真履行党风廉政建设职责，严肃查处腐败案件，扎实开展廉洁自律警示教育，大力纠正征地拆迁、退耕还林补助粮款政策兑现、环境污染等方面关系群众利益的突出问题，促进了廉政建设和政风建设。着力建设平安陇南，扎实推进社会治安防控体系建设，加强基层司法所、派出所和法庭建设，开展“万人下基层排查矛盾纠纷”活动，依法打击各类刑事犯罪，进一步加强信访工作，维护了社会大局稳定。坚持凝聚人和，激发内部活力，全市人民的凝聚力、创造力不断增强，万众一心、众志成城、政通人和、共谋发展的团结和谐局面不断巩固。

各位代表，2007年，我们全面完成了各项任务，开创了经济社会发展的新局面。这些成绩的取得，是省委、省政府关怀支持的结果，是市委正确领导、科学决策的结果，是市人大、市政协和社会各界支持帮助的结果，是全市人民共同艰苦努力奋斗的结果。在这里，我代表市政府，向奋战在各条战线上的劳动者，向驻陇南人民解放军、武警官兵和中央、省驻陇南单位，向各民主党派、工商联、无党派爱国人士、离退休老干部，向所有关心支持陇南发展进步的各界人士表示衷心的感谢和崇高的敬意！

一年工作的实践和取得的成效给予我们许多有益的启示，我们深切体会到，要把加快陇南科学发展的伟大事业不断推向前进，早日实现富民强市的目标，必须始终坚持在动态中审视市情，准确把握陇南发展的整体性特点和阶段性特征，坚定不移地走具有中国特色、时代特征和陇南特点的发展之路；必须突出比较优势，扬长避短，化劣为优，把特色优势与国内国际两个市场对接，把比较优势转化为市场需求，把特色优势转化成经济优势，把特色产业做到最大化；必须始终坚持把解放思想、改革开放作为动力源泉，破除一切阻碍陇南发展的思想观念和体制机制；必须始终牢记欠发达的基本市情，树立长期艰苦奋斗的思想，发扬“人一之、我十之，人十之、我百之”的甘肃精神和在龙昌公路建设实践中铸就的昌河坝精神，越是条件艰苦，越要付出超常的努力，越要自力更生、艰苦奋斗，永葆自强不息、坚韧不拔、百折不挠、顽强拼搏的精神品格，依靠自己的智慧和力量改变贫穷落后面貌；必须始终坚持把立足点放在依靠人民群众上，做到一切为了陇南人民，一切依靠陇南人民，一切发展成果由陇南人民共享，把人民群众建设美好家园的积极性充分调动起来，形成全民创业的强大力量，共同致力于建设生态陇南、文化陇南、和谐陇南、富庶陇南的伟大实践。

各位代表，陇南的发展正在发生深刻变化，我们陇南市必强、陇南人必富的信念比过去任何时候都更坚定。但我们清醒地认识到，发展不足、发展不够、发展不快，经济总量小，质量不高，贫困面大、贫困程度深是我们主要的矛盾和问题，与全国、全省的发展差距仍然很大，欠发达仍然是我们的基本市情，前进中还面临不少困难和问题。主要是：农业基础条件差，投入水平低，综合效益还不高；农业产业化程度低，工业和农产品加工链条短，特色产业还没有做到最大化，资源优势还没有变成经济优势；招商引资的办法不多，力度不大，环境不优，招商引资的整体水平不高；社会事业发展仍显滞后，群众行路、饮水、上学、看病、住房等方面的困难仍然较多；社会管理、城市管理薄弱，有的县在城市建设上没有大的突破，经营管理赶不上，严重存在脏乱差问题；商贸流通发展比较缓慢，市场建设和经营监管严重滞后，马路市场现象十分突出。政府自身建设需要进一步加强，驾驭市场经济的能力不强，办事效率、落实程度、工作质量仍然不高，一些干部思想不够解放，责任心、事业心不强，干事创业的激情、干劲和开拓创新能力不足，抓工作落实的责任、水平还有很大差距，大手大脚、胡花乱用、奢侈浪费、消极腐败现象在一些地方和部门仍然存在。对这些问题，我们将在今后的工作中采取有效措施认真加以解决。

二、2008年全市经济社会发展的

主要任务和工作重点

2008年是全面贯彻落实党的十七大精神和省十一次党代会精神的关键一年，是全面贯彻市第二次党代会精神，推动陇南经济社会发展上台阶的重要一年。我们要倍加努力，奋发有为，乘势而上，再创佳绩，把全市经济社会不断推向新的发展阶段。

今年全市经济社会发展的总体思路是：以党的十七大精神为指导，用科学发展观统领经济社会发展全局，全面贯彻省十一次党代会精神和市第二次党代会做出的各项战略部署，突出科学发展的主题与和谐共享的发展方向，坚定不移地实施举特色旗、走富民路、建小康市的发展战略，坚定不移地推进“一纲九目”的工作重点，坚定不移地落实“四个突破口”的工作要求，坚定不移地实现“两个高于、五个大幅度增长”的经济发展目标，坚定不移地为人民群众多办实事好事，坚持全党抓项目、突出抓特色、重点抓工业、增长抓转变、增收抓调整的经济工作总方针，奋力推动全市经济社会又好又快发展。

奋斗目标是：全市生产总值增长13%，达到132亿元；全社会固定资产投资增长41%，达到70.9亿元；大口径财政收入确保增长26%，突破20亿元大关，力争增长30%；市县级财政收入确保增长30%，达到7.5亿元，力争增长40%，突破8亿元；规模以上工业增加值增长21%，达到37.5亿元；社会消费品零售总额增长15%，达到28.7亿元；城镇居民人均可支配收入增长8.5%，达到8300元；农民人均纯收入净增150元，达到1750元；物价指数控制在104.2左右；新增城镇就业人数1.14万人，城镇登记失业率控制在3.9%以内；人口自然增长率控制在6.8‰以内；万元生产总值能耗下降4%；二氧化硫排放总量控制在0.88万吨以内;化学需氧量控制在0.87万吨以内。

实现上述奋斗目标，必须坚定不移地落实“一纲九目”，始终坚持以发展抓项目为总纲，进一步强化各级领导包抓项目“五个一”责任制，力争在项目储备、争取、实施上实现大突破，全年储备项目1000个，实施投资50万元以上的项目1000项以上，重点抓好武都至罐子沟段高速公路、兰渝铁路、支线机场、尖凡隧道、礼武公路、橙子沟电站、沙湾电站等重大项目。以超常的举措抓招商引资，不断创新招商引资方式，组织小分队常年在外开展招商，下决心整治招商引资和项目建设环境，吸引更多的投资者到陇南投资兴业，力争全年签约招商引资项目200项以上，签约资金90亿元以上，到位资金35亿元以上，项目履约率达到75%。拓宽抓项目的领域，既争取政策性项目，又重视市场性项目；既抓经济建设项目，又抓社会事业项目；既抓产业开发项目，又抓基础设施项目；既抓支撑财政增收的全局性重点项目，又抓关系民生的项目；既抓好大项目，又抓好中小项目，再建成一批带动经济结构调整、拉动经济社会发展、对财政增收有支撑作用的大项目、好项目。

围绕发展抓项目这个总纲，我们将着力抓好九个方面的工作：

（一）坚持扩张规模与提质增效并重，努力实现农业特色产业最大化。高度重视“三农”工作，进一步强化农业基础地位，切实加大对农业的投入，加快农业结构调整步伐。大力发展以特色产业为主体的现代农业，因地制宜，发挥优势，既抓核桃、花椒、中药材、畜牧养殖等数县一品、全市一品的优势主导产业，又抓油橄榄、茶叶、桔子、无花果等区域性、独一性的特色产业。核桃、花椒产业尽快实现适宜区全覆盖，今年补植新栽核桃100万亩，新发展花椒40万亩，油橄榄5万亩，茶叶5万亩，当年种植中药材53万亩，种植蔬菜50万亩，出栏商品牛羊50万头（只），银杏、苹果、蚕桑等产业面积进一步扩张，水面养殖、花卉开发实现新突破。兴办一批起点高、规模大、带动力强的龙头加工企业，申报省级和国家级龙头企业3户、1户。加快农产品交易市场建设，完善农产品市场体系，拓宽农产品营销渠道。大力提高农业科技含量，重点抓好全膜双垄沟播新技术的推广，今年坚决完成6万亩的种植任务。加强农业服务体系建设，深入实施“五个千”到村工程，开展农民科技培训42万人，发展科技示范户5000户，创办农民技术学校136所，发展农民专业合作经济组织123个。特色产业研究院尽快挂牌启动，充分发挥其在农业特色产业科技研究、推广和应用方面的作用。继续抓好“一注册三认证”工作，建立农产品质量安全监管体系，推进农业标准化生产进程。加大农业综合开发力度，提高农业综合效益，在白龙江、白水江川坝河谷地带建设农业特色产业开发万元田示范区，西汉水流域建成农业特色产业综合开发示范区，嘉陵江流域建成高效优质农业特色产业示范区，全市建成“万元田”10万亩、“多千田”45万亩，千方百计增加农民收入。

（二）坚持重点抓工业，全力实现工业发展重大突破。围绕加快培育有色冶金、水电、绿色食品、建筑建材、医药化工、制造业、加工业、旅游工艺品等八大主导产业，进一步扩大工业经济总量，提高工业对经济的贡献率，着力建设成州10万吨锌冶炼技改、宝徽集团11万吨锌冶炼、徽县10万吨铅冶炼、武都水泥厂技改、阳山金矿开发等重点工业项目，加快“三江一水”梯级电站建设，新上文县屈家河口，宕昌沙湾等水电站项目，继续抓好文县苗家坝、麒麟寺电站，武都大园坝、汉王电站等续建项目。坚持引进新技术、新工艺，改造提升传统产业，大力发展制造业、绿色食品加工工业、石材工业等后续接替产业。加强资本运作和资产重组，做大做强企业集团，力争独一味公司早日上市，宝徽、成州集团积极创造条件，争取尽早上市。坚持实施民营主体战略，放手发展非公有制经济，非公有制经济增加值增长速度达到20%。进一步规范矿产资源开发秩序，加大矿产资源整合力度，推进矿产资源采、选、冶科学开发。高度重视环境建设，召开环境保护大会，加强能源资源节约和生态环境保护，加大环保执法力度，实施“三江一水”环境综合治理，继续抓好矿区污染企业和高耗能企业环境污染治理，坚决打击偷开、偷排等违法行为。深入推进节能减排工作，加强重点用能企业管理，坚决淘汰落后生产能力，通过技术改造推进节能降耗。大力发展循环经济，积极开展矿产资源、固体废物、水资源综合利用和再生资源循环利用。

（三）以公路交通建设为重点，全面加快基础设施建设。发扬不畏艰险、顽强拼搏，众志成城、艰苦奋斗，不等不靠、以干克难，勇于创新、敢于胜利的昌河坝精神，深入持久地开展公路交通大建设，着力改变陇南交通滞后的状况。完成通乡四级油路449公里，通乡及通村等级公路2152公里，建成联网路、出口路18条236.5公里，打通成县至略阳、两当至天水、宕昌南河至腊子口等出口路；昌河坝公路康县、西和段铺油硬化。省上确定的通乡、通村公路项目全部开工建设，为了保证质量，加快建设进度，今后的通乡油路建设，由各县（区）上报，市上对路基工程统一组织验收，验收通过后再安排铺油。全力抓好武都至罐子沟段高速公路、礼武公路建设的征地拆迁工作，支持212线宕昌段公路改造，创造良好建设环境；争取兰渝铁路上半年开工建设；加快支线机场前期工作。加强能源建设，抓紧实施武都330、西和330千伏送变电工程，全面完成农村电网改造一、二期节余工程和西部完善工程；抓好饮水安全、梯田建设、灌溉工程、病险水库除险加固工程和以长治工程为重点的小流域综合治理，年内新修梯田8万亩；新增有效灌溉面积3.6万亩，发展集雨补灌面积4万亩，节水灌溉面积4万亩。加强生态文明建设，大力实施退耕还林、天然林保护、公益林建设、自然保护区建设、公路行道树绿化，加快120万亩生态林工程建设，今年完成40万亩。强化土地和水资源保护，严格执行开发建设项目环境保护、水土保持“三同时”制度，加强泥石流灾害综合治理，推进生态自然修复工程，建立生态补偿机制。加强通讯建设，抓好移动、电信网络和村村通电话、移动村村通工程，年内基本实现村村通电话，扩大移动信号覆盖面。

（四）提升旅游产业开发整体水平，带动第三产业快速发展。积极落实三省十二方旅游区域合作协议，推进陕甘川毗邻地区大旅游经济圈建设。完成重点景区建设性详规编制，加强以景区道路、通讯、宾馆饭店为主的旅游基础设施建设，提升十大旅游精品景区的服务档次和接待水平，再创建和申报一批3A、4A级景区和星级宾馆；抓好旅游产品开发，提高旅游商品收入在旅游总收入中的比重；组建旅游公司和旅游开发协会，加强旅游人才队伍建设，提高旅游管理服务水平；不断创新旅游宣传推介，多方开辟客源市场，力争全年接待旅游人数280万人次，旅游综合收入达到4亿元，使旅游产业跃上新台阶。发挥旅游业对第三产业的辐射带动作用，推动交通运输、餐饮娱乐、宾馆饭店、房地产开发等传统服务业和连锁经营、电子商务、物流配送、金融服务、要素市场、会展经济等新型服务业快速发展。

（五）加快城镇建设步伐，大力提升城镇化水平。按照“一年一变样、三年大变样”的要求，推进市区、八县县城、建制镇建设，加快城镇化进程。一是加快市区建设。重点抓好市区长江大道、莲湖文化广场、皇庙街拆迁改造、盘旋路西南商住小区开发、陇南农产品交易市场、市区垃圾处理场、污水处理配套管网工程等项目，市区长江大道旧城区段元月底前建成通车，东江新区段和钟楼滩段国庆节前全线贯通，如期建成东江新区路网管网、东江水大桥、北峪河大桥、建民大桥，全面启动建设行政办公楼、商业小区、商品住宅楼，尽早动工建设武都城区集中供暖供气工程，实现市区面积翻一番。二是加快八县县城建设。尽快完成八县县城建设详规编制；坚持一手抓旧城改造、一手抓新城拓展，徽县、两当继续加快城市改造建设的步伐，在建设经营管理上带好头，走在前面。今年成县、西和、礼县、文县、康县、宕昌要在城市建设上有新举措、新突破、新变化。重点抓好供水扩建、电网改造、集中供热和污水处理管网配套工程建设，扩大城镇规模，完善城镇功能，发挥辐射带动作用。制定优惠政策，鼓励有条件的富裕农民向城镇转移。三是加快建制镇建设。有重点地选择一批区位优、实力强、后劲足、辐射广的建制镇，高标准地搞好规划和建设，使建制镇建设取得较大突破。进一步强化全市城市规划管理，严格按规划推进城镇建设；创新城市建设投融资体制，建立多元化的投入机制；坚持依法拆迁、和谐拆迁，依法统征土地，着力解决拆迁难、土地征用难的问题；加强城市综合管理，积极创建文明城市，大力提升城市品味，创造良好人居环境。

（六）举全市之力实施扶贫开发，扎实推进新农村建设。坚定不移地走好产业开发、整村推进、劳务输转、移民搬迁、科技扶贫五条路子，探索建立贫困村信用扶持机制，用足用好扶贫贴息政策，实现扶贫工作由输血式、救济性扶贫向扎根性、造血型扶贫转变，年内减少贫困人口8.5万人。继续实施整村、整片、整流域整体推进，实施整村推进105个，抓好以工代赈项目、易地扶贫搬迁项目和库区移民项目的实施，完成移民2.16万人；大力发展劳务经济，推动劳务输出由体力型向技能型、由分散短期输出向有组织长期稳定转出转变，提高劳务输转质量效益，培训农民工15.2万人，输转劳务60万人，创劳务收入21亿元。全面启动西汉水流域扶贫综合开发。进一步强化扶贫帮扶责任，深入开展结穷亲、解民忧、帮民富、受教育、长德才、求发展、促和谐帮扶活动，多为群众办实事、办好事。

扎实推进新农村建设，按照“因地制宜，整体规划，分类指导，分步实施”的原则，高标准、高质量地抓好新农村试点村建设，培育一批具有较强示范辐射带动作用的新农村建设典型。在发展生产的基础上，着力整治村容村貌，解决脏乱差问题，改善落后的生活习惯，倡导文明生活新风尚。对这项工作各县区要列入重要议事日程，专题研究，拿出具体实施意见，着手着力推动，务必取得实效。全面推进以乡村道路、清洁能源、改水、改房、改炉、改灶、改厕、改圈为重点的农村基础设施建设，加快发展农村教育、卫生、文化、科技等社会事业，解决群众行路、吃水、用电、上学、就医、通讯等实际问题，组织引导农民绿化村庄、美化住宅、净化家园，促进社会文明进步。

（七）加强财政工作，进一步搞活商贸流通。坚持围绕财政增收抓经济，大力培植财源税源，依法加强税收征管，强化非税收入管理，确保财政收入持续大幅增长。优化财政支出结构，优先保证职工工资、扶贫开发、特色产业、改善民生、社会事业、重点项目建设等方面的支出。加强财政支出管理，严格预算约束，建立财政资金使用跟踪问效制度，坚持勤俭办一切事业，把可用的财力统筹起来，集中财力办大事，提高财政资金使用效益。

坚持内贸外贸齐抓，努力繁荣市场，扩大消费，拉动经济增长。加快商品流通体系市场建设，培育壮大市场营销企业，促进商贸流通，发展现代物流，构建区域性、综合性及专业性大市场，加快消费市场和农产品批发市场建设，建成12个大型超市。鼓励城乡居民扩大住房、汽车、通信、教育、体育、文化等方面的消费，培育新的消费热点。充分利用文化、旅游、民间传统节会，积极开展各种商品展销活动。加强市场规范管理，加快农贸市场建设，积极开展市场专项整治，下决心解决以路为市的问题，这项工作今年必须取得重大突破。大力发展外向型经济，培育出口创汇特色产业，重点培育奶花芸豆、核桃、中药材、苹果、大蒜、肉牛等出口基地，各县区都要建成2户以上的出口创汇企业，都要有出口创汇产品，外贸出口今年增长50%，对外贸易力争达到3000万美元。

（八）进一步深化各项改革，全方位扩大对外开放。深化国有企业改革，做好已改制企业的后续完善工作，有条件的企业逐步建立现代企业制度。健全国有资产监管体系，加强对企业国有资本运营及其收益的监督管理。深化财政金融体制改革，推进县乡财政体制和“乡财县管”体制改革，完善部门预算制度。强化金融监管，进一步完善金融服务体系和社会信用体系，充分发挥金融对地方经济发展的推动作用。深化农村综合改革，推进乡镇机构改革，认真实施种子管理体制、农业科技服务体系和动物防疫体制改革，建立适应市场经济体制的农牧业服务体系。深化投融资体制改革，强化市场融资手段，发挥城投公司、中小企业担保中心等融资机构的作用；规范企业投资项目核准制和备案制，实行政府投资公益性项目“代建制”。深化人事制度改革，坚持党政机关和参照管理事业单位“凡进必考”，严格执行事业单位公开招聘制度。加快推进教育人事制度改革和内部管理体制改革，深化科技、文化体制改革，增强社会事业发展活力。

以更大的气魄推进对外开放，坚持“引进来”与“走出去”相结合，拓宽对外开放领域，提高对外开放水平。抓住东部沿海发达地区产业、资本向中西部转移的机遇，扩大利用外资、招商引资的规模。广泛开展科技、教育、文化、艺术等方面的交流，进一步加强与省内外高等院校、科研院所的联系与合作，积极参加国内和省内的重要节会，继续筹备办好旅游节等各类节会，更多地吸引和聚集各种生产要素。

（九）加快发展社会事业，推动和谐陇南建设。在加快经济发展的基础上，更加注重社会建设，更加注重改善民生，努力使全市人民学有所教、劳有所得、病有所医、老有所养、住有所居，共享改革发展成果。坚持教育优先发展，努力办人民满意的教育。进一步增加教育投入，巩固义务教育，大力发展高中教育、职业技术教育和幼儿教育。加快实施农村初中校舍危房改造项目，抓好宕昌县“两基”达标，年内全市整体实现“两基”目标。建成一批省、市级示范学校。开工建设陇南一中新校区。筹建陇南市职教中心。大力支持陇南师专更快发展。加强教师队伍建设，不断创新教学方式，全面提高教育教学质量。大力发展文化事业，推动文化大发展大繁荣。加快博物馆、文化馆、图书馆、乡镇文化站、“农家书屋”等公益性文化基础设施建设；大力挖掘陇南独特的地域文化，做好文物古迹和非物质文化遗产的保护、开发和利用；实施重大文化产业项目带动战略，加快新闻出版、广播影视、互联网络、文化创意产业发展；繁荣文学艺术创作，推出一批有影响力的精品力作。建成陇南人民广播电台，积极筹建市广播电视大厦，推进数字电视整体转换。加强城乡社区体育设施建设，建成100个全民健身工程点，广泛开展“迎奥运”全民健身活动，办好陇南市第七届体育运动会，提高竞技体育水平。提升科技创新能力，发挥科技对经济社会发展的支撑作用。召开全市科技发展大会，强化以企业为主体的自主创新体系建设，建成省级技术创新中心1个、市级企业技术研发中心10个，培育技术创新示范企业和科技示范企业10户，力争申请专利30件，实施好省、市、县列科技项目，获得科技成果55项。推进科技特派员基层创业，培训农村科技明白人10万人以上，科技入户率达到90%。不断完善医疗卫生体系，提高人民群众健康水平。召开全市卫生发展大会，制定加快卫生事业发展的政策措施。加强县乡卫生基础设施建设，新建扩建85所县乡医疗机构和城市社区卫生服务机构，建立覆盖城乡的卫生服务体系。加强医德医风建设，大力提高医疗服务质量。强化食品药品市场监管，保障群众饮食用药安全。全面落实人口和计划生育各项政策措施，稳定低生育水平，提高出生人口素质，综合治理出生人口性别比偏高问题。实施积极的就业政策，全面推进就业再就业。激发全民创业活力，以创业带动就业。大力发展劳动密集型产业、服务业和各类中小企业，增加就业岗位，多渠道扩大就业。健全面向全体劳动者的职业技能培训制度，加大对困难群众的就业援助力度，统筹做好城镇新增劳动力就业、下岗失业人员再就业、城市零就业家庭成员就业和农村富余劳动力转移就业工作。加强对高等院校毕业生的就业指导和服务，鼓励和引导高等院校毕业生面向农村、面向基层就业。加快建立城乡社会保障体系，保障人民群众基本生活。不断完善城市居民最低生活保障制度，做到动态管理下的应保尽保。确保城乡保障配套资金及时足额到位。全面实施城镇居民基本医疗保险，使医疗保险制度基本覆盖城镇全体居民。进一步扩大城镇社会保险覆盖面，推进养老、失业、医疗、工伤保险基本覆盖个体工商户和各种所有制企业的进程。加强社会福利公益项目建设，力争年内建成市社会福利院、儿童福利院、流浪儿童救护中心。加强社会管理，维护安定团结的社会局面。完善应急管理体制机制，提高保障公共安全和处置突发事件的能力。加强城市社区建设，充实社区保障机构工作力量，增强社区服务管理功能。强化安全生产监督管理体系建设，严格落实各级安全生产责任，坚决遏制重特大安全事故。完善社会治安防控体系，继续抓好派出所、司法所、人民法庭等基层基础建设，加强社会治安综合治理，依法防范和打击违法犯罪活动，深入开展平安陇南创建活动。切实做好人民群众来信来访工作，落实领导接访包案责任制和领导信访接待日制度，拓宽社情民意表达渠道，有效预防和化解各类社会矛盾，把问题解决在基层、解决在萌芽状态。加强食品等基本生活必需品和农资产品价格监控。抓好民族宗教工作，依法加强宗教事务管理。坚持开展全民国防教育，做好双拥共建工作，巩固和发展军政军民团结，落实省国防动员委员会军事需求总体计划，完成兵员征集任务，发展老龄、残疾人和关心下一代事业，维护妇女儿童合法权益。

各位代表，市委、市政府着眼于解决广大人民群众最迫切、最现实、最关心的利益问题，提高人民群众的物质文化生活水平，决定今年要办好修建100座便民大桥、3000公里乡村公路；改造1000户困难户房屋；建成沼气池2万口、发展太阳灶5万台；解决10万人的饮水安全问题；建立村干部离职养老保险补贴制度；提高新型农村合作医疗参合农民补贴补助标准；农村居民最低生活保障标准提高10%；将30万城乡低收入困难群众纳入低保范围；建立建筑行业农民工工资保证金制度；为17220户城市低收入家庭发放廉租住房补贴、新建经济适用房10万平方米；全面落实中小学“两免一补”政策，为47万中小学生免除学杂费、课本费，为6.7万寄宿制学生发放生活补助；新增100个通有线电视的乡镇、建成10万户广播电视“村村通”工程；加快少数民族乡村经济社会发展，年内实现4个少数民族乡通油路；建成8个中心敬老院、100个“五保家园”；建立乡镇民政工作办公室等16件实事。确定了每一件实事的包抓领导、责任单位。我们将倾注全力办好这些实事，使人民群众得到更多实惠。

三、加强政府自身建设

各位代表，全面落实2008年的各项工作任务，推动经济社会发展上新台阶，政府肩负着重大责任。我们要更好地履行职责，就必须大力加强政府自身建设，加快建设服务政府、法治政府、效能政府、廉洁政府、务实政府。

一是坚持加强学习，努力提高执政能力和执政水平。把深入学习贯彻党的十七大精神作为首要政治任务，坚持用党的理论创新的最新成果武装头脑，指导实践，推动工作，进一步增强贯彻落实科学发展观的自觉性和坚定性，把科学发展观贯穿于陇南经济社会发展的各个领域、各个方面。刻苦学习市场经济、法律、科技、金融、社会管理等方面的知识，努力提高驾驭市场经济的能力，依法行政的能力，管理社会事务的能力，协调利益关系的能力，增强开展群众工作的本领，激发社会活力的本领，处理人民内部矛盾的本领，维护社会稳定的本领。

二是强化服务职能，不断优化服务环境。坚持一切为了发展、一切服从发展、一切服务于发展，进一步强化政府服务职能，打造行为规范、便捷高效、公正诚信、群众满意的服务环境，为人民群众和各类市场主体提供良好服务。深入开展学习型、竞争型、务实型、创新型、清廉型“五型”机关创建活动，着力解决服务意识差、服务效率低等突出问题，大力提升行政效能。进一步减少行政审批事项，规范行政审批行为，下决心简化办事环节和办事程序，推行“一站式”、“一条龙”服务等方便快捷的办事方式，全面落实首问责任制、服务承诺制、限时办结制，对行政不作为、行政乱作为，严格实行行政问责，对造成重大损失和恶劣影响的，依法依纪严肃处理。

三是坚持依法行政，推进依法治市进程。加强法制宣传教育，扎实开展“五五”普法，提高干部群众法治意识和法律素质。严格按照法定权限和程序行使权力、履行职责，依法有序推进各项工作。进一步加强和改进行政执法，加大对规范市场经济秩序、资源环境保护、与人民群众生活密切相关的法律法规的执法力度，为人民群众安居乐业提供有力的法治保障和法律服务。自觉接受人大法律监督、政协民主监督、新闻舆论监督和社会公众监督，认真落实市人大及其常委会的各项决议、决定和市政协的建议，不断提高人大代表建议和政协委员提案的办理质量，支持监察、审计部门依法履行监督职责。扩大政务公开的范围和层次，保障公民的知情权、参与权、表达权、监督权。完善村务公开、厂务公开制度，扩大基层民主。

四是强化宗旨意识，全心全意为人民谋福祉。我们的根基在人民，血脉在人民，力量在人民，任何时候任何情况下与人民群众同呼吸共命运的立场不能变，全心全意为人民服务的宗旨不能忘，始终把人民的呼声当作第一信号，把人民的需要当作第一选择，把人民的利益当作第一考虑，把人民满意当作第一标准，心里始终装着群众，时刻把群众的冷暖挂在心上，把心思用在群众身上，把功夫下在群众身上，把政绩体现在群众身上，多深入基层，深入群众，听民声，察民情，解民忧，集民智，帮民富，多办利长远、打基础的事，多办顺应民意、为民谋利的事，多办化解矛盾、增进和谐的事，真心实意地为群众办实事、做好事、解难事，维护好、实现好、发展好人民群众的根本利益。

五是切实改进工作作风，扑下身子抓落实。突出抓落实这个关键点和着力点，增强对党的路线方针政策的执行力和对市委重大决策部署的落实力。继续实行领导干部抓项目抓产业“五个一”责任制，联系乡镇、贫困村、企业、学校的“四个一”制度。建立严格的督查制度，对重点工作实行每月督查、每季分析、半年检查、年终考核。坚决落实会议减少三分之一、文件减少三分之一、领导干部下基层的时间不少于三分之一的要求，用更多的时间、精力谋大事、抓大事、办实事。树立只争朝夕、雷厉风行的良好作风，克服效率低下、节奏缓慢，坚决杜绝推诿扯皮，敷衍塞责，抓而不紧，抓而不实。领导干部要带头改进作风抓落实，带着对群众的深厚感情抓落实，到困难最多的地方去，到矛盾最多的地方去，到工作推不开的地方去，到条件最艰苦的地方去，打开工作局面，努力创造经得起群众、实践和历史检验的工作业绩。

六是加强廉政建设，树立为民务实清廉的良好形象。坚持从严治政，认真履行廉政建设责任制，扎实推进惩治和预防腐败体系建设。深入开展廉洁自律警示教育，严肃查处违法违纪案件，认真解决教育、医疗、土地征用、房屋拆迁、安全生产、环境污染、退耕还林政策兑现等方面关系群众利益的突出问题。拓展从源头上预防和治理腐败的领域，进一步完善建设工程招投标、经营性土地使用权出让、政府采购、产权交易、领导干部任期经济责任审计等制度。领导干部要严格廉洁自律，自觉接受监督，作清正廉洁的表率。

各位代表，加快科学发展，构建和谐陇南，是我们的光荣使命和神圣职责。我们要在市委的坚强领导下，在市人大、市政协的监督支持下，紧紧依靠全市280万人民，大力发扬“人一之、我十之，人十之、我百之”的甘肃精神，脚踏实地，艰苦创业，埋头苦干，狠抓落实，全面超额完成今年的各项工作任务，把陇南经济社会推向新的发展阶段。
